# Trends in Birth Rates After Elimination of Cost Sharing for Contraception by the Patient Protection and Affordable Care Act

**DOI:** 10.1001/jamanetworkopen.2020.24398

**Published:** 2020-11-06

**Authors:** Vanessa K. Dalton, Michelle H. Moniz, Martha J. Bailey, Lindsay K. Admon, Giselle E. Kolenic, Anca Tilea, A. Mark Fendrick

**Affiliations:** 1 Department of Obstetrics and Gynecology, University of Michigan, Ann Arbor; 2 Institute for Healthcare Policy and Innovation, University of Michigan, Ann Arbor; 3 Program on Women’s Healthcare Effectiveness Research, University of Michigan, Ann Arbor; 4 Institute for Social Research Population Studies Center, University of Michigan, Ann Arbor; 5 Department of Internal Medicine, University of Michigan, Ann Arbor; 6 Center for Value-Based Insurance Design, University of Michigan, Ann Arbor

## Abstract

**Importance:**

Reducing out-of-pocket costs is associated with improved patterns of contraception use. It is unknown whether reducing out-of-pocket costs is associated with fewer births.

**Objective:**

To evaluate changes in birth rates by income level among commercially insured women before (2008-2013) and after (2014-2018) the elimination of cost sharing for contraception under the Patient Protection and Affordable Care Act (ACA).

**Design, Setting, and Participants:**

This cross-sectional study used data from Clinformatics Data Mart database from January 1, 2008, to December 31, 2018, for women aged 15 to 45 years who were enrolled in an employer-based health plan and had pregnancy benefits for at least 1 year. Women without household income information and women with evidence of having undergone a hysterectomy were excluded.

**Exposure:**

Section 2713 of the ACA.

**Main Outcomes and Measures:**

The primary outcome was the proportion of reproductive-aged women with a live birth by year (measured yearly from 2008 to 2018 [11 time points]) within 3 income categories. The secondary outcome was the distribution of contraceptive method fills in 3 categories by year: (1) most effective methods (long-acting reversible contraception or sterilization), (2) moderately effective methods (pill, patch, ring, and injectable), and (3) no prescription or surgical method.

**Results:**

The analytic sample included 4 590 989 women (mean [SD] age; 30.8 [9.1] years in 2013; 3 069 053 White [66.9%]) enrolled in 47 721 health plans. A total of 500 898 participants (40.8%) resided in households with incomes less than 400% of the federal poverty level in 2013. In all 3 years (2008, 2013, and 2018), women in the lowest income category were younger than women in the other income groups (median range, 21-22 years vs 30-34 years) and in households with a higher median number of dependents (9-10 vs 2-4). There was an associated decrease in births in all income groups in the period after the elimination of out-of-pocket costs. The estimated probability of birth decreased most precipitously among women in the lowest income group from 8.0% (95% CI, 7.4%-8.5%) in 2014 to 6.2% (95% CI, 5.7%-6.7%) in 2018, representing a 22.2% decrease (*P* < .001). The estimated probability decreased in the middle income group by 9.4%, from 6.4% (95% CI, 6.3%-6.4%) to 5.8% (95% CI, 5.7%-5.8%) (*P* < .001), and in the highest income group by 1.8%, from 5.6% (95% CI, 5.6%-5.7%) to 5.5% (95% CI, 5.4%-5.5%) (*P* < .001) in the period after the elimination of cost sharing.

**Conclusions and Relevance:**

In this cross-sectional study, the elimination of cost sharing for contraception under the ACA was associated with improvements in contraceptive method prescription fills and a decrease in births among commercially insured women. Women with low income had more precipitous decreases than women with higher income, suggesting that enhanced access to contraception may address well-documented income-related disparities in unintended birth rates.

## Key Points

**Question:**

Was the elimination of cost sharing for contraception under the Patient Protection and Affordable Care Act associated with a change in birth rates among women in different income groups?

**Findings:**

In this cross-sectional study, the estimated probability of birth decreased most precipitously among women in the lowest income group from 8.0% in 2014 to 6.2% in 2018. The probability of a birth also decreased significantly among women in higher income groups, but this decrease was smaller in magnitude.

**Meaning:**

These findings suggest that contraception insurance coverage without consumer cost sharing may be associated with decreased income-related disparities in unintended birth rates.

## Teaser

This cross-sectional study assesses the association of the elimination of cost sharing for contraception under the Patient and Protection and Affordable Care Act (ACA) with birth rates by income level among commercially insured women.

## Introduction

Nearly half of pregnancies in the US are unplanned, and marked income-related disparities in unintended pregnancy rates are well described.^1^ Unintended pregnancies are associated with delayed prenatal care, reduced likelihood of breastfeeding, maternal depression, physical violence, and higher maternal and infant mortality rates.^2,3^ Undesired pregnancies have societal costs, including an estimated $5 billion per year in direct and indirect costs for the US health care system.^4^ On the basis of evidence that contraception is effective at preventing pregnancy, cost-effective, and consistent with Healthy People 2020 goals, the Institute of Medicine identified contraception as an essential preventive health service for women.^5^ As a result, contraception was included on the list of preventive care services required to be covered without consumer cost sharing by most insurers under §2713 of the Patient Protection and Affordable Care Act (ACA).^6^

Studies have consistently shown that removal of out-of-pocket costs (OOPCs) for contraception is associated with increased consistency of use, method continuation, and selection of the most effective methods.^7,8,9,10,11^ The ACA effectively eliminated cost sharing for most commercially insured women by 2014, and its implementation was associated with increased use of prescription contraception, particularly the use of long-acting reversible contraception (LARC) (eg, intrauterine device or implant).^8,9,10,12^ It remains unknown, however, whether these changes in contraceptive use were associated with fewer births. Furthermore, it is also unknown whether this policy might specifically benefit women with low income, who may be more sensitive to prices and also experience higher rates of unintended births compared with women with higher income.^1^ Accordingly, our objective was to examine changes in birth rates by income level among commercially insured women before (2008-2013) and after (2014-2018) the elimination of cost sharing for contraception under the ACA.

## Methods

This cross-sectional study used deidentified patient data from Clinformatics Data Mart (Optum) to examine the association of the elimination of OOPCs with contraceptive provision patterns and births among commercially insured women. The Clinformatics Data Mart database includes patient demographic information, including household income and data on patient cost sharing. Race/ethnicity is included in this sample and is populated using a combination of sources including self-report, public records, and demographic derivations. This study was deemed exempt by the University of Michigan institutional review board. Informed consent was waived because this was a retrospective review of existing deidentified data. This study followed the Strengthening the Reporting of Observational Studies in Epidemiology (STROBE) reporting guideline.

The analytic sample was drawn from a population of 7 761 568 women aged 15 to 45 years enrolled in employer-based health plans between January 1, 2008, and December 31, 2018. We restricted the sample to women with continuous enrollment in a single employer-based health plan with pregnancy benefits for at least 1 year. Women without household income information and women with evidence of having undergone a hysterectomy were excluded. The sample selection is described in detail elsewhere^9^ (eFigure in the Supplement). All outcomes and patient characteristics were collected at the woman-year level.

Our hypothesis was that the removal of financial barriers (ie, OOPCs) to contraception would be associated with a greater proportion of women using either moderately effective or the most effective contraceptive methods, all of which require a prescription or a procedure and can be costly to initiate. We believed that improved contraception use patterns would be associated with a decrease in births because a large proportion of births follow an unintended conception. Previous work reported that OOPCs decreased rapidly after mandated first dollar coverage was implemented in similar health plans.^8,10,13,14^ The preperiod (2008-2013) and postperiod (2014-2018) that we chose were based on observed decreases in OOPCs for LARC methods because they were known to have the highest level of OOPCs at baseline.^9,13^

Our primary outcome was the proportion of commercially insured women with a birth by income level between 2008 and 2018. Information on household income and number of covered dependents was included in our data source. Household income for the primary beneficiary was demographically derived at the household level. Yearly guidelines for poverty-level computations from the US Department of Health and Human Service’s Office of the Assistant Secretary for Planning and Evaluation^15^ were used with the household income and number of covered dependents data to categorize women into 3 income groups relative to the federal poverty level (FPL): less than 100% FPL; 100% to 399% FPL; and greater than or equal to 400% FPL.

Our secondary outcome, contraceptive fill patterns, was derived to provide additional evidence that either supported or refuted our hypothesis. We assessed whether there were parallel trends in contraception fill patterns consistent with observed changes in births. We did not consider contraception fill patterns as a primary outcome because changes in contraception use after the implementation of the ACA have been reported previously.^9,10,13,14^ Although incidence of use (eg, LARC placements, sterilization procedures) are reliably identified in claims, estimating prevalence of use is less accurate for long-acting methods, especially sterilization. We categorized contraceptive method fill patterns by effectiveness: (1) most effective methods (LARC or sterilization), (2) moderately effective methods (pill, patch, ring, or injectable), and (3) no prescription or surgical method. Method fills were identified using device, procedure, diagnosis, and pharmacy codes (eTable 1 in the Supplement). Long-acting or sterilization methods were identified using *International Classification of Diseases, Ninth Revision*, *International Statistical Classification of Diseases and Related Health Problems, Tenth Revision*, and *Current Procedural Terminology, Fourth Edition* (Healthcare Common Procedure Coding System) codes. Both procedure and device codes were required to indicate LARC use. Pharmacy fills in a given calendar year were used to identify moderately effective method users. Women with evidence of more than 1 method category fill in a year (eg, pills, sterilization) were categorized in the most effective method category for that calendar year.

### Statistical Analysis

We evaluated changes in births before (2008-2013) and after (2014-2018) the elimination of OOPCs for contraception under the ACA by household income category (<100% FPL, 100%-399% FPL, and ≥400% FPL) with a comparative interrupted time-series model conducted at the woman-year level.^16^ This design approach compares outcomes before and after an intervention while accounting for trends over time. Our comparative interrupted time-series approach expanded on the standard interrupted time-series design with the inclusion of comparison groups based on household income. The interactions among time, household income category, and the elimination of OOPCs were of primary interest within the comparative interrupted time-series framework, and the following interactions were included in our model: time by elimination of OOPC, time by household income, elimination of OOPC and household income, and time by household income by elimination of OOPC. The comparative interrupted time-series model was fit with a generalized estimating equation to account for repeated observations per woman over the study period and controlled for age, race/ethnicity, number of dependents, geographic region, and health plan type. Model-based adjusted predictions were obtained. Birth trends and levels before and after the elimination of OOPCs for contraception were compared within and between household income groups. Similar models and methods assessed contraceptive fill patterns. All statistical tests were 2-sided, and an α level of .05 was used to determine statistical significance. Claims data management was performed in SAS, version 9.2 (SAS Institute), and statistical analyses were conducted in Stata, version 14.1 (StataCorp).

## Results

Our analytic sample included 4 590 989 unique women (mean [SD] age, 30.8 [9.1] years in 2013) enrolled in 47 721 health plans between 2008 and 2018. Women were predominately White (3 069 053 [66.9%]), and 500 898 of the participants in 2013 (40.8%) resided in households with incomes below 400% FPL (Table). Compared with women in the higher income groups, women in the lowest income category were younger (median range, 21-22 years vs 30-34 years), resided in households with a larger number of dependents (median range, 9-10 vs 2-4), and were more racially diverse (Black proportion range, 16.9%-25.3% vs 6.7%-19.2%). In this sample, the percentage of LARC placements that incurred OOPCs decreased rapidly from 66.3% (11 304 of 17 039) in 2008 to 6.7% (1845 of 27 426) in 2014 and 3.2% (973 of 29 954) in 2018. During the study period, we identified 766 087 births among 20 455 355 observations (woman-years).

**Table.** Patient-Level Characteristics of the Study Sample

Characteristic	Women by FPL^a^								
	2008			2013			2018		
	<100% (n = 2693)	100%-399% (n = 402 545)	≥400% (n = 772 723)	<100% (n = 4154)	100%-399% (n = 496 744)	≥400% (n = 728 387)	<100% (n = 4371)	100%-399% (n = 500 633)	≥400% (n = 456 620)
Age, median (IQR), y	21 (17-36)	31 (22-38)	34 (26-40)	22 (18-34)	30 (21-38)	32 (24-39)	22 (19-32)	30 (21-38)	33 (25-39)
Dependents, median (IQR), No.	10 (9-11)	4 (2-5)	3 (2-4)	9 (9-10)	4 (3-5)	3 (2-4)	9 (8-10)	4 (3-5)	2 (1-4)
Delivered in claim year	8.2 (7.2-9.4)	6.3 (6.2-6.4)	6.0 (5.9-6.0)	9.2 (8.3-10.1)	6.5 (6.4-6.6)	5.6 (5.6-5.7)	6.5 (5.8-7.3)	5.9 (5.8-5.9)	5.6 (5.5-5.6)
Race/ethnicity									
White	47.8 (45.9-49.7)	57.1 (57.0-57.3)	71.7 (71.6-71.8)	53.3 (51.8-54.9)	60.9 (60.7-61.0)	73.3 (73.2-73.4)	52.8 (51.3-54.3)	61.8 (61.6-61.9)	69.7 (69.6-69.8)
Black	25.3 (23.7-27.0)	19.2 (19.1-19.3)	8.2 (8.1-8.2)	21.4 (20.1-22.6)	17.0 (16.9-17.1)	7.4 (7.3-7.4)	16.9 (15.8-18.0)	12.0 (11.9-12.1)	6.7 (6.6-6.7)
Hispanic	20.9 (19.4-22.5)	18.1 (18.0-18.2)	10.3 (10.2-10.4)	17.5 (16.4-18.7)	16.2 (16.1-16.3)	9.5 (9.4-9.6)	20.1 (19.0-21.4)	16.4 (16.3-16.5)	10.8 (10.7-10.9)
Asian	4.7 (4.0-5.6)	3.6 (3.5-3.7)	6.9 (6.8-6.9)	6.1 (5.4-6.9)	4.2 (4.1-4.2)	7.3 (7.3-7.4)	4.9 (4.3-5.6)	4.5 (4.5-4.6)	7.6 (7.5-7.7)
Unknown or missing	1.4 (1.0-1.9)	2.0 (1.9-2.0)	2.9 (2.9-3.0)	1.7 (1.3-2.10)	1.8 (1.8-1.8)	2.5 (2.5-2.5)	5.3 (4.7-6.0)	5.3 (5.3-5.4)	5.2 (5.2-5.3)
Insurance plan									
POS	60.6 (58.7-62.5)	62.1 (61.9-62.2)	65.6 (65.4-65.7)	74.2 (72.8-75.5)	74.3 (74.1-74.4)	76.2 (76.1-76.3)	73.4 (72.1-74.7)	75.8 (75.7-75.9)	77.1 (77.0-77.2)
EPO or HMO	34.4 (32.6-36.2)	33.6 (33.5-33.8)	30.8 (30.7-30.9)	23.5 (22.2-24.8)	23.8 (23.7-23.9)	21.9 (21.8-22.0)	24.5 (23.2-25.8)	22.2 (22.1-22.4)	21.1 (21.0-21.3)
PPO	5.0 (4.2-5.9)	62.1 (61.9-62.2)	3.5 (3.5-3.6)	2.2 (1.8-2.7)	1.7 (1.7-1.8)	1.7 (1.6-1.7)	1.8 (1.4-2.2)	1.4 (1.4-1.5)	1.1 (1.1-1.2)
Indemnity or other	0 (0-0.2)	0.1 (0.1-0.1)	0.1 (0.1-0.1)	0.2 (0.1-0.4)	0.2 (0.2-0.2)	0.3 (0.2-0.3)	0.4 (0.2-0.6)	0.5 (0.5-0.5)	0.6 (0.6-0.7)
Region									
Southeast	48.2 (46.3-50.1)	49.8 (49.7-50.0)	44.9 (44.8-45.0)	45.9 (44.4-47.4)	46.3 (46.1-46.4)	42.0 (41.9-42.2)	41.8 (40.3-43.3)	41.7 (41.6-41.8)	40.8 (40.7-40.9)
Great Lakes or Northern Plains	23.5 (21.9-25.2)	21.3 (21.2-21.5)	21.6 (21.5-21.7)	27.1 (25.8-28.5)	25.3 (25.1-25.4)	24.9 (24.8-25.0)	26.0 (24.7-27.3)	26.7 (26.6-26.8)	24.1 (24.0-24.3)
Pacific	12.9 (11.7-14.3)	12.4 (12.3-12.5)	13.2 (13.2-13.3)	9.0 (8.2-9.9)	10.5 (10.4-10.5)	11.9 (11.8-11.9)	9.6 (8.8-10.5)	11.2 (11.1-11.2)	12.9 (12.8-13.0)
Northeast	5.1 (4.3-6.0)	8.3 (8.2-8.4)	12.6 (12.5-12.6)	5.5 (4.8-6.3)	8.5 (8.4-8.5)	12.7 (12.6-12.8)	4.7 (4.1-5.4)	9.3 (9.2-9.4)	13.1 (13.0-13.2)
Mountain	9.2 (8.1-10.4)	7.6 (7.5-7.7)	7.4 (7.3-7.4)	11.6 (10.6-12.6)	9.3 (9.3-9.4)	8.4 (8.3-8.4)	17.6 (16.5-18.8)	11.0 (10.9-11.1)	8.9 (8.8-9.0)
Unknown	1.1 (0.8-1.6)	0.5 (0.5-0.6)	0.3 (0.2-0.3)	0.9 (0.6-1.2)	0.2 (0.2-0.3)	0.2 (0.1-0.2)	0.3 (0.2-0.5)	0.2 (0.1-0.2)	0.1 (0.1-0.1)
Service use									
Preventive care office visit	24.9 (23.3-26.6)	37.5 (37.3-37.6)	49.4 (49.3-49.5)	29.3 (27.9-30.7)	42.0 (41.9-42.2)	53.2 (53.0-53.3)	30.1 (28.7-31.5)	44.8 (44.7-44.9)	53.4 (53.3-53.5)
LARC insertion	1.3 (0.9-1.8)	1.5 (1.5-1.6)	1.4 (1.4-1.4)	2.3 (1.9-2.8)	2.3 (2.3-2.4)	2.1 (2.1-2.2)	3.2 (2.7-3.7)	3.2 (3.1-3.2)	3.1 (3.0-3.1)
Proportion with 0 cost sharing^b^									
Preventive care office visit	35.2 (31.6-39.0)	31.7 (31.4-31.9)	31.1 (30.9-31.2)	92.4 (90.7-93.8)	94.4 (94.3-94.5)	85.4 (95.3-95.4)	98.3 (97.5-99.0)	98.6 (98.5-98.6)	98.8 (98.7-98.8)
LARC insertion	37.1 (21.5-55.1)	32.4 (31.2-33.6)	34.4 (33.5-35.3)	84.5 (75.8-91.1)	85.9 (85.3-86.5)	87.4 (86.9-87.9)	96.4 (91.8-98.8)	96.6 (96.3-96.8)	97.0 (96.7-97.3)

Abbreviations: EPO, exclusive provider organization; FPL, federal poverty level; HMO, health maintenance organization; IQR, interquartile range; LARC, long-acting reversible contraception; POS, point of service; PPO, preferred provider organization.

^a^ Data are presented as percentage (95% CI) unless otherwise indicated.

^b^ Among service users only.

**Figure 1.**
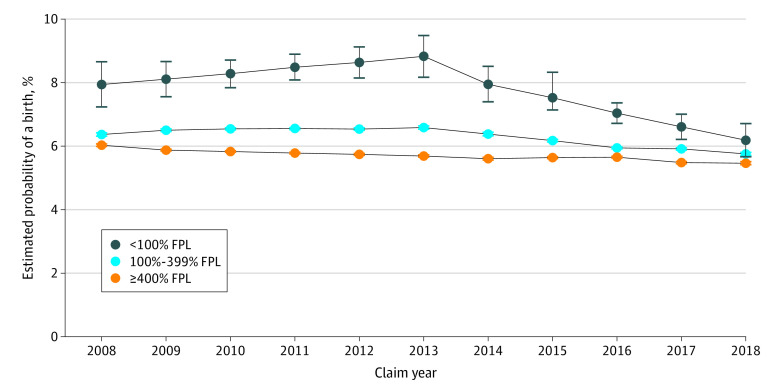
Estimated Probability of a Birth for Women Aged 15 to 45 Years Between 2008 and 2018 Error bars represent 95% CIs. FPL indicates federal poverty level.

There was a significantly associated decrease in births in all income groups in the period after the elimination of OOPCs (Figure 1). The estimated probability of birth decreased most precipitously among women in the lowest income group from 8.0% (95% CI, 7.4%-8.5%) in 2014 to 6.2% (95% CI, 5.7%-6.7%) in 2018, representing a 22.2% decrease. The estimated probability also decreased in the middle income group by 9.4%, from 6.4% (95% CI, 6.3%-6.4%) to 5.8% (95% CI, 5.7%-5.8%), and in the highest income group by 1.8%, from 5.6% (95% CI, 5.6%-5.7%) to 5.5% (95% CI, 5.4%-5.5%) after the elimination of OOPCs (eTable 2 in the Supplement). Within each income group, annual birth rates before and after the elimination of OOPCs differed significantly. For example, the change from 2013 to 2014 in the less than 100% FPL group was –0.0087 (95% CI, –0.017 to –0.0003), in the 100% to 399% FPL group was –0.002 (95% CI, –0.0028 to –0.001), and in the 400% or greater FPL group was –0.0008 (95% CI, –0.001 to –0.0003). Annual birth rates after the elimination of OOPCs also differed significantly among the household income groups (<100% FPL vs 100%-399% FPL, 0.0032 [95% CI, 0.0009-0.005]; <100% FPL vs ≥400% FPL, 0.0054 [95% CI, 0.003-0.008]; 100%-399% FPL vs ≥400% FPL, 0.0022 [95% CI, 0.0019-0.0025]). Birth rates decreased more rapidly in low and middle income categories than in the high income group (Figure 1). Differences in birth rates between women in the highest and lowest income groups decreased by 62.2% between 2008 and 2018.

Changes in contraception fill patterns between 2008 and 2018 were consistent with the observed changes in birth rates. Within each income group, trends in annual rates of not filling a prescription method differed significantly before and after the elimination of OOPCs in 2014 (<100% FPL, –0.007 [95% CI, –0.012 to –0.002]; 100%-399% FPL, –0.009 [95% CI, –0.0097 to –0.0088]; ≥400% FPL, –0.008 [95% CI, –0.0084 to –0.0075]).

**Figure 2.**
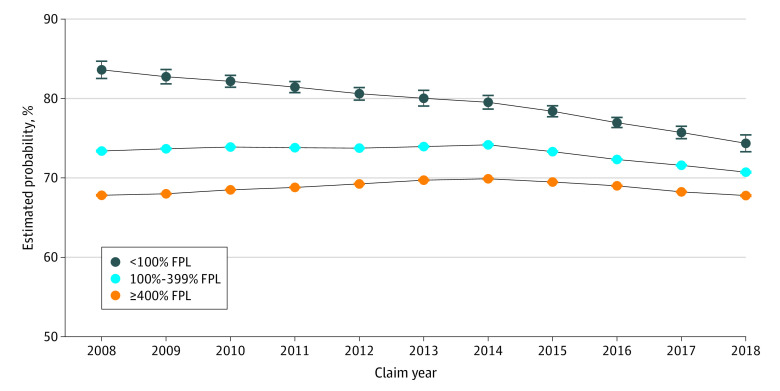
Estimated Probability of Women Aged 15 to 45 Years Without Evidence of Prescription Contraception Fill Between 2008 and 2018 Error bars represent 95% CIs. FPL indicates federal poverty level.

**Figure 3.**
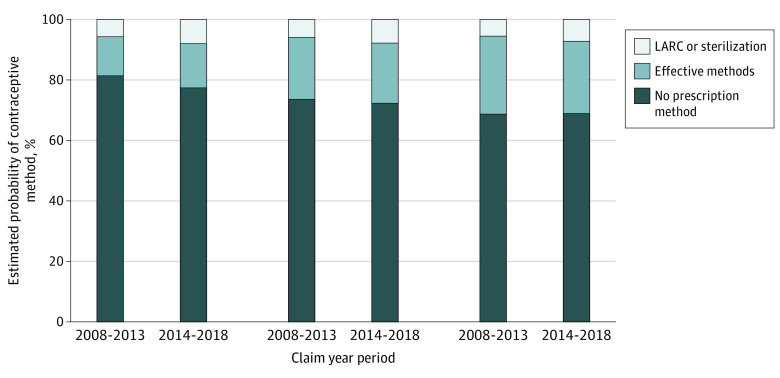
Adjusted Contraception Method Fill by Year and Income Category Women with evidence of more than 1 method category in a year (eg, pills and sterilization) were categorized in the effective method category. LARC indicates long-acting reversible contraception.

Trends in annual rates of not filling a prescription method of contraception after 2014 also differed significantly across income groups, with the 2 lower household income groups demonstrating a more rapid decrease than the higher income group (Figure 2 and eTable 3 in the Supplement). Conversely, shifts in adjusted prescription contraception method fills in the higher income group were largely between different categories of prescription methods (eg, pills to LARC) (Figure 3).

## Discussion

Results from our study of this large sample of commercially insured women suggest that the elimination of cost sharing was associated with an increase in the birth rate reduction within all income groups but most notably in the 2 lower income groups. These observed trends were associated with a smaller difference in birth rates between women in the highest and lowest income categories. Observed changes in contraceptive method fills were consistent with changes in births. Trends in prescription contraception fills, especially for the most effective methods, were positively associated with the elimination of cost sharing. Furthermore, the observed differences in method fills across income groups were consistent with our hypothesis that women with low income may be more sensitive to the price of contraception. These findings suggest that the elimination of cost sharing for contraception may be associated not only with improved contraception use patterns but also with fewer births. Furthermore, the observed differences in birth rates across income groups suggest that expanded coverage of prescription contraception may be associated with a reduction in income-related disparities in unintended pregnancy rates.

Reducing unintended pregnancies has been a public health goal in the US for decades because there are serious consequences for women, families, and society. In 2011, 42% of unintended pregnancies (excluding miscarriages) ended in abortion^1^ and two-thirds of unplanned births were funded by public insurance programs such as Medicaid.^17^ There are also serious long-term consequences of mistimed births for women,^18,19,20,21,22,23^ for the life opportunities of their children,^21,22,23,24^ and for society. Removing cost-related barriers for birth control (via the ACA mandate or otherwise) alone may not eliminate unintended pregnancy, but it may be an important component of the multifaceted approach needed to address this important public health concern.

Contraception is a clinically efficacious and cost-effective strategy for reducing unintended pregnancy. A policy that eliminates OOPCs for contraception is consistent with our clinical goals of ensuring that all individuals can decide whether and when to have children. This policy may also be an effective strategy to address well-documented income-related disparities in unintended pregnancy rates in the US. Recent court decisions, including the 2020 Supreme Court decision upholding the rules that expand exemptions from the contraceptive mandate,^25^ could roll back improvements in access for some women. We believe that continued monitoring is warranted.

### Strengths and Limitations

This study has strengths. These data offer advantages not available from other sources, including the size of the sample and information on household income.

This study also has limitations. First, this study could not establish a causal relationship between the elimination of cost sharing or changes in contraception use and decreases in births. However, it adds to a growing body of evidence that consistently concludes that the elimination of cost sharing is associated with improved contraceptive use patterns.^9,10,13,14^ Changes in the population of women covered by employer health plans might also explain the observed decrease in births. Furthermore, in administrative data, we were not able to observe services or procedures without some level of insurance coverage or services obtained outside the member’s health plan. Because we could not directly measure pregnancy intention, we assumed that changes in contraception use patterns reflected pregnancy desires and that decreases in births represented unwanted pregnancies averted. In addition, we cannot generalize our findings to all commercially insured women or to women in general. This limitation might be especially true of the lowest income group in the sample because they appeared to have unique characteristics.

## Conclusions

In this cross-sectional study, the elimination of cost sharing for contraception under the ACA was associated with improvements in contraceptive method prescription fills and a decrease in births among commercially insured women. Women with low income had more precipitous decreases than women with higher income, suggesting that enhanced access to contraception may address well-documented income-related disparities in unintended birth rates.

## Notes

Supplement. **eFigure.** Analytic Sample Derived From 7 761 568 Reproductive-Aged Women, 2008-2018 **eTable 1.** Codes Used to Identify Births and Contraceptive Treatment Patterns **eTable 2.** Model Results for Figure 1 Generation **eTable 3.** Model Results for Figure 2 Generation

## References

[1] Finer LB, Zolna MR Declines in unintended pregnancy in the United States, 2008-2011. N Engl J Med. 2016;374(9):843-852. doi:10.1056/NEJMsa1506575 26962904 PMC4861155

[2] Brown SS, Eisenberg L The Best Intentions: Unintended Pregnancy and the Well-being of Children and Families. National Academy Press; 1995. 25121228

[3] Gipson JD, Koenig MA, Hindin MJ The effects of unintended pregnancy on infant, child, and parental health: a review of the literature. Stud Fam Plann. 2008;39(1):18-38. doi:10.1111/j.1728-4465.2008.00148.x 18540521

[4] Sonfield A, Kost K, Gold RB, Finer LB The public costs of births resulting from unintended pregnancies: national and state-level estimates. Perspect Sex Reprod Health. 2011;43(2):94-102. doi:10.1363/4309411 21651708

[5] Institute of Medicine Committee on Preventive Services for Women Clinical Preventive Services for Women: Closing the Gaps. The National Academies Press; 2011.

[6] Kaiser Family Foundation Preventive services covered by private health plans under the Affordable Care Act. Accessed October 1, 2020. https://kff.org/health-reform/fact-sheet/preventive-services-covered-by-private-health-plans/

[7] Liang SY, Grossman D, Phillips KA Women’s out-of-pocket expenditures and dispensing patterns for oral contraceptive pills between 1996 and 2006. Contraception. 2011;83(6):528-536. doi:10.1016/j.contraception.2010.09.013 21570550

[8] Heisel E, Kolenic GE, Moniz MM, Intrauterine device insertion before and after mandated health care coverage: the importance of baseline costs. Obstet Gynecol. 2018;131(5):843-849. doi:10.1097/AOG.0000000000002567 29630013

[9] Dalton VK, Carlos RC, Kolenic GE, The impact of cost sharing on women’s use of annual examinations and effective contraception. Am J Obstet Gynecol. 2018;219(1):93.e1-93.e13. doi:10.1016/j.ajog.2018.04.051 29752935

[10] Carlin CS, Fertig AR, Dowd BE Affordable Care Act’s mandate eliminating contraceptive cost sharing influenced choices of women with employer coverage. Health Aff (Millwood). 2016;35(9):1608-1615. doi:10.1377/hlthaff.2015.1457 27605640

[11] Postlethwaite D, Trussell J, Zoolakis A, Shabear R, Petitti D A comparison of contraceptive procurement pre- and post-benefit change. Contraception. 2007;76(5):360-365. doi:10.1016/j.contraception.2007.07.006 17963860

[12] Becker NV The impact of insurance coverage on utilization of prescription contraceptives: evidence from the Affordable Care Act. J Policy Anal Manage. 2018;37(3):571-601. doi:10.1002/pam.22064 29993229

[13] Pace LE, Dusetzina SB, Fendrick AM, Keating NL, Dalton VK The impact of out-of-pocket costs on the use of intrauterine contraception among women with employer-sponsored insurance. Med Care. 2013;51(11):959-963. doi:10.1097/MLR.0b013e3182a97b5d 24036995 PMC6702955

[14] Pace LE, Dusetzina SB, Keating NL Early impact of the Affordable Care Act on uptake of long-acting reversible contraceptive methods. Med Care. 2016;54(9):811-817. doi:10.1097/MLR.0000000000000551 27213549 PMC4982821

[15] US Department of Health and Human Services Office of the Assistant Secretary for Planning and Evaluation Poverty guidelines computations. Accessed August 10, 2020. https://aspe.hhs.gov/

[16] Kim Y, Steiner P Quasi-experimental designs for causal inference. Educ Psychol. 2016;51(3-4):395-405. doi:10.1080/00461520.2016.1207177 30100637 PMC6086368

[17] Sonfield A, Kost K Public costs from unintended pregnancies and the role of public insurance programs in paying for pregnancy-related care: national and state estimates for 2010. Accessed August 13, 2020. https://www.guttmacher.org/report/public-costs-unintended-pregnancies-and-role-public-insurance-programs-paying-pregnancy

[18] Bailey MJ More power to the pill: the impact of contraceptive freedom on women's lifecycle labor supply. Q J Econ. 2006;121(1):289-320. doi:10.1093/qje/121.1.289 PMC11929136 40124738

[19] Goldin C, Katz LF The power of the pill: oral contraceptives and women's career and marriage decisions. J Polit Econ. 2002;110(4):730-770. doi:10.1086/340778

[20] Hotz VJ, McElroy SW, Sanders SG Teenage childbearing and its life cycle consequences: exploiting a natural experiment. J Hum Resour. 2005;XL(3):683-715. doi:10.3368/jhr.XL.3.683

[21] Bailey MJ, Malkova O, McLaren ZM Do family planning programs increase children’s opportunities? evidence from the war on poverty and the early years of Title X. J Hum Resour. 2019;54(4):825-856. doi:10.3368/jhr.54.4.1216-8401R1 31768076 PMC6876122

[22] Bailey MJ Fifty years of family planning: new evidence on the long-run effects of increasing access to contraception. Accessed August 13, 2020. https://www.brookings.edu/bpea-articles/fifty-years-of-family-planning-new-evidence-on-the-long-run-effects-of-increasing-access-to-contraception/10.1353/eca.2013.0001 PMC4203450 25339778

[23] Stevenson AJ, Genadek KR, Yeatman S, Mollborn S, Menken J The educational impact of expanded contraceptive access. Accessed August 13, 2020. http://paa2019.populationassociation.org/uploads/191070

[24] Ananat EO, Hungerman DM The power of the pill for the next generation: oral contraception’s effects on fertility, abortion, and maternal & child characteristics. Rev Econ Stat. 2012;94(1):37-51. doi:10.1162/REST_a_00230 22389533 PMC3289404

[25] Supreme Court of the United States Blog. *Little Sisters of the Poor Saints Peter and Paul Home v. Pennsylvania* Accessed August 18, 2020. https://www.scotusblog.com/case-files/cases/little-sisters-of-the-poor-saints-peter-and-paul-home-v-pennsylvania/

