# Supplementary material for: Trends in Birth Rates After Elimination of Cost Sharing for Contraception by the Patient Protection and Affordable Care Act
Source: JAMA Netw Open. 2020 Nov 6;3(11):e2024398. doi: 10.1001/jamanetworkopen.2020.24398 (PMC7648257; doi:10.1001/jamanetworkopen.2020.24398)
Supplement: Supplement. — eFigure. Analytic Sample Derived From 7 761 568 Reproductive-Aged Women, 2008-2018 eTable 1. Codes Used to Identify Births and Contraceptive Treatment Patterns eTable 2. Model Results for Figure 1 Generation eTable 3. Model Results for Figure 2 Generation [file jamanetwopen-e2024398-s001.pdf]

Supplementary Online Content

Dalton VK, Moniz MH, Baily MJ, et al. Trends in birth rates after elimination of cost sharing for contraception by the Patient Protection and Affordable Care Act. *JAMA Netw Open*. 2020;3(11):e2024398. doi:10.1001/jamanetworkopen.2020.24398

eFigure. Analytic Sample Derived From 7 761 568 Reproductive-Aged Women, 2008-2018

eTable 1. Codes Used to Identify Births and Contraceptive Treatment Patterns

eTable 2. Model Results for Figure 1 Generation

eTable 3. Model Results for Figure 2 Generation

This supplementary material has been provided by the authors to give readers additional information about their work.

© 2020 Dalton VK et al. *JAMA Network Open.*

eFigure. Analytic Sample Derived From 7 761 568 Reproductive-Aged Women, 2008-2018

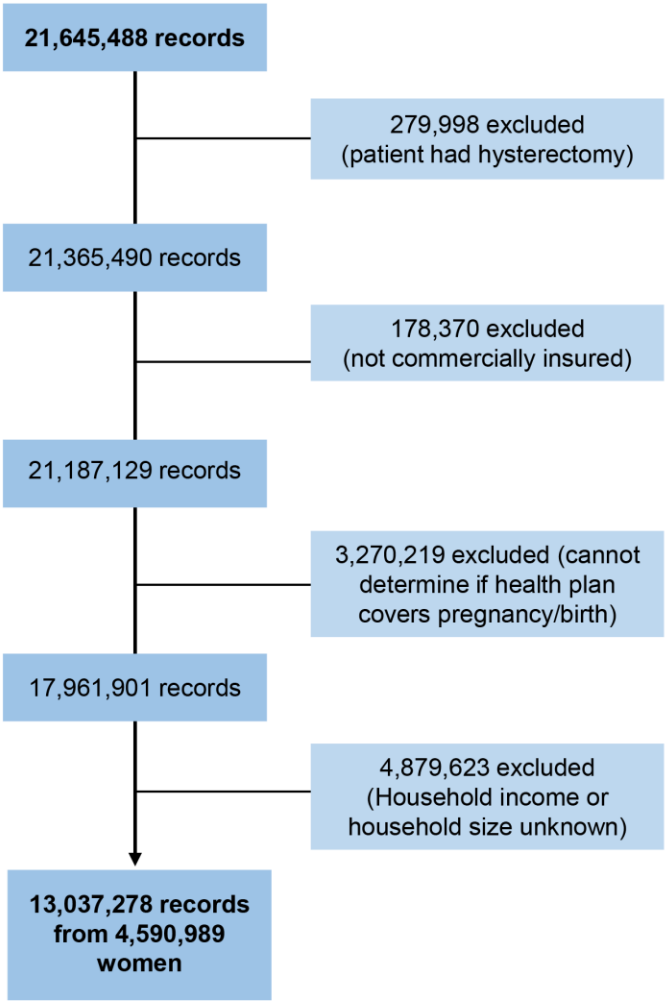

© 2020 Dalton VK et al. *JAMA Network Open.*

eTable 1. Codes Used to Identify Births and Contraceptive Treatment Patterns

Deliveries

	**ICD-9 Dx**	**ICD-10 Dx**
Include	V27, V27.0, V27.1, V27.2, V27.3, V27.4, V27.5, V27.6, V27.7, V27.9, 650	Z37, Z37.0, Z37.1, Z37.2, Z37.3, Z37.4, Z37.5, Z37.59, Z37.6, Z37.69, Z37.7, Z37.9, 080
Exclude	630, 631, 632, 633, 633.0, 633.00, 633.01, 633.1, 633.10, 633.11, 633.2, 633.20, 633.21, 633.8, 633.80, 633.81, 633.9, 633.90, 633.91, 634, 634.x, 635, 635.x, 636, 636.x, 637, 637.x, 638.x, 639, 639.0, 639.1, 639.2, 639.3, 639.4, 639.5, 639.6, 639.8, 639.9, V24, V24.0, V24.1, V24.2	A34, O00, O00.0, O00.00, O00, O00.1, O00.2, O00.8, O00.9, O01.9, O02, O02.1, O03, O03.0, O03.1, O03.2, O03.30, O03.31, O03.32, O03.33, O03.34, O03.34, O03.39, O03.4, O03.5, O03.6, O03.7, O03.8x, O03.9, O04.5, O04.6, O04.7, O04.8x, O07.0, O07.1, O07.2, O07.3x, O07.4, O08.0, O08.1, O08.2, O08.3, O08.4, O08.5, O08.6, O08.7, O08.81, O08.83, O08.89, O08.9, Z33.2, Z39, Z39.0, Z39.1, Z39.2
	**ICD-9 Px**	**ICD-10 Px**
Include	72, 72.x, 73, 73.01, 73.09, 73.1, 73.2, 73.21, 73.22, 73.3, 73.4, 73.5, 73.51, 73.59, 73.6, 73.8, 73.9, 73.92, 73.93, 73.94, 73.99, 74, 74.1, 74.2, 74.4, 74.9, 74.99	0Q820ZZ, 0Q823ZZ, 0Q824ZZ, 0Q830ZZ, 0Q833ZZ, 0Q834ZZ, 0U7C7ZZ, 0W8NXZZ, 10A07ZZ, 10A08ZZ, 10D00Z0, 10D00Z1, 10D00Z2, 10D07Z3, 10D07Z4, 10D07Z5, 10D07Z6, 10D07Z7, 10D07Z8, 10E0XZZ, 10J07ZZ, 10S07ZZ, 10S0XZZ, 10900ZC, 10903ZC, 10904ZC, 10907ZA, 10907ZC, 10908ZA, 10908ZC, 3E030VJ, 3E033VJ, 3E040VJ, 3E043VJ, 3E050VJ, 3E053VJ, 3E060VJ, 3E063VJ, 3E0DXGC, 3E0P7GC
Exclude	69.01, 69.51, 74.91, 75.0	N/A
	**DRG**	**CPT**
Include	370, 371, 372, 373, 374, 375<br>765, 766, 767, 768, 774, 775, 766	59400, 59409, 59410, 59610,59612, 59614, 59510, 59514, 59515, 59618, 59620, 59622

IUD

	**ICD-9 Dx**	**ICD-10 Dx**
Include	V25.11	Z30.430
	**ICD-10 Px**	
Include	0UH97HZ, 0UH98HZ, 0UHC7HZ, 0UHC8HZ, 0UH90HZ	
	**NDC**	
Include	50419042101, 50419042201, 51285020401, 50419042208, 50419042271, 50419042301, 51285020402, 52544003554, 00023585801, 50419042401, 50419042408, 50419042471, 50419042308	
	**HCPCS**	**CPT**
Include	J7297, J7298, J7300, J7301, J7302, Q0090, S4981, S4989	58300

Implant

	**ICD-9 Dx**	**ICD-10 Dx**
Include	V25.5	Z30.8
	**NDC**	
Include	00052027201, 00052027401, 00052433001, 00052027480	
	**HCPCS**	**CPT**
Include	J7306, J7307	11981

Sterilization

	**ICD-9 Dx**	**ICD-10 Dx**
Include	V25.2	Z30.2

© 2020 Dalton VK et al. *JAMA Network Open.*

	ICD-9 Px	ICD-10 Px
Include	66.2	0U574ZZ, 0U578ZZ, 0UL74CZ, 0UL74DZ, 0UL74ZZ, 0UL78DZ, 0UL78ZZ
	**HCPCS**	**CPT**
Include	A4264	58565, 58600, 58605, 58611, 58615, 58670, 58671

Other Hormonal Methods

	ICD-9 Dx	ICD-10 Dx
Include	V25.01, V25.41	Z30.011, Z30.013, Z30.015, Z30.016, Z30.41, Z30.42, Z30.44, Z30.45
	**NDC**	
Include	00009074630, 00009074635, 00009470901, 00009470913, 00009737604, 00009737607, 00009737611, 00247210801, 00703680101, 00703680104, 00703681121, 23490585401, 54569370100, 54569490400, 54569552700, 54569561600, 54569621900, 54868361300, 54868410000, 54868410001, 54868525700, 55045350501, 59762453701, 59762453702, 59762453801, 59762453802, 59762453809, 00009062601, 52125064001, 52125091501, 54868334801, 68788923301, 00008111720, 00008111730, 00008251402, 00008253505, 00008253601, 00008253605, 00052026106, 00052028306, 00062125100, 00062125115, 00062125120, 00062133220, 00062141116, 00062141123, 00062171400, 00062171415, 00062176100, 00062176115, 00062178100, 00062178115, 00062179600, 00062179615, 00062190120, 00062190320, 00062190700, 00062190715, 00062191000, 00062191015, 00247052028, 00247069028, 00247069128, 00247069228, 00247139828, 00247151328, 00247151628, 00247151728, 00247176404, 00247176421, 00247176521, 00247198621, 00247198628, 00247200828, 00247201004, 00247201008, 00247201028, 00247201228, 00247201328, 00247214728, 00247216928, 00247217028, 00247223028, 00247223528, 00247226028, 00247226828, 00378655053, 00378727253, 00378729253, 00430042014, 00430048214, 00430053014, 00430053550, 00430057014, 00430057045, 00430058014, 00430058045, 00430058114, 00430058514, 00430058545, 00555034458, 00555071558, 00555900867, 00555900942, 00555901058, 00555901258, 00555901467, 00555901658, 00555901858, 00555902058, 00555902542, 00555902557, 00555902658, 00555902742, 00555902757, 00555902858, 00555903270, 00555903458, 00555904358, 00555904558, 00555904758, 00555904958, 00555905058, 00555905158, 00555905167, 00555906458, 00555906467, 00555906558, 00555906658, 00555906667, 00555912366, 00555913167, 00555913179, 00603359017, 00603359049, 00603752117, 00603752149, 00603752517, 00603752549, 00603754017, 00603754049, 00603760615, 00603760648, 00603760715, 00603760748, 00603760817, 00603760917, 00603762517, 00603762549, 00603763417, 00603763449, 00603764017, 00603764217, 00603766317, 00603766517, 23490765301, 23490767001, 23490769901, 24090080184, 24090096184, 35356001468, 35356001568, 35356002168, 35356025528, 35356037028, 43386062030, 45802084054, 50419040201, 50419040203, 50419040303, 50419040503, 50419040701, 50419040703, 50419041112, 50419041128, 50419043306, 50419043312, 50452025115, 50458017115, 50458017615, 50458017815, 50458019115, 50458019411, 50458019416, 50458019615, 50458019715, 50458025115, 51285005866, 51285007997, 51285008070, 51285008198, 51285008297, 51285008370, 51285008498, 51285008787, 51285009158, 51285009287, 51285011458, 51285043165, 51285054628, 51285076993, 51285094288, 51285094388, 52544014331, 52544017572, 52544020431, 52544021028, 52544021928, 52544022829, 52544023528, 52544023531, 52544024531, 52544024728, 52544024828, 52544025428, 52544025928, 52544025988, 52544026528, 52544026531, 52544026829, 52544026884, 52544027428, 52544027431, 52544027536, 52544027928, 52544028754, 52544029128, 52544029231, 52544029241, 52544029528, 52544038328, 52544038428, 52544047536, 52544055028, 52544055228, 52544055428, 52544062928, 52544063028, 52544063128, 52544084728, 52544084828, 52544089228, 52544093628, 52544094028, 52544094928, 52544095021, 52544095121, 52544095328, 52544095428, 52544095931, 52544096691, 52544096728, 52544098131, 52544098231, 52959045002, 54569067900, 54569068500, 54569068501, 54569068900, 54569068901, 54569143900, 54569384400, 54569422200, 54569422201, 54569426900, 54569427301, 54569481700, 54569487800, 54569487801, 54569489000, 54569498400, 54569499700, 54569499800, 54569516100, 54569534300, 54569534900, 54569549300, 54569549302, 54569579600, 54569579700, 54569579800, 54569581600, 54569582600, 54569603200, 54569612800, 54569614400, 54569614500, 54569627200, 54569628000, 54569628100, 54868042800, 54868044300, 54868050200, 54868050700, 54868050801, 54868050901, 54868051600, 54868151200, 54868156400, 54868231600, 54868260600, 54868270100, 54868377200, 54868386300, 54868394800, 54868409300, 54868423900, 54868436900, 54868453800, 54868459000, 54868460700, 54868473000, 54868473100, 54868474200, 54868474500,	

© 2020 Dalton VK et al. *JAMA Network Open.*

	54868475400, 54868477600, 54868481400, 54868482800, 54868485100, 54868486000, 54868491100, 54868502800, 54868528600, 54868532600, 54868535600, 54868582600, 54868582800, 54868594200, 55045283902, 55045348506, 55045349701, 55045349801, 55045378106, 55045378206, 55045378302, 55289024708, 55289088704, 55887005228, 55887028628, 58016474701, 58016482701, 66993061128, 66993061528, 68180084313, 68180084413, 68180084613, 68180084813, 68180085413, 68180087611, 68180087613, 68180089713, 68180089813, 68180090213, 68462030329, 68462030529, 68462030929, 68462031629, 68462031829, 68462038829, 68462039429, 68462055629, 68462056529, 68462063729, 68462064693, 00052026108, 00093313491, 00093614891, 00378655056, 00378727753, 00378728098, 00378728398, 00378728590, 00378728756, 00378729656, 00378729853, 00378730053, 00430042060, 00430042095, 00430048295, 00430053060, 00430053095, 00430057060, 00781407515, 00781410352, 00781557515, 16714034001, 16714034002, 16714034003, 16714034601, 16714034602, 16714034603, 16714034802, 16714034803, 16714035901, 16714035902, 16714036001, 16714036002, 16714036003, 16714036301, 16714036302, 16714036303, 16714036501, 16714036502, 16714036503, 16714036603, 16714037001, 16714037002, 16714037004, 16714040701, 16714040702, 16714040704, 16714041601, 16714041602, 16714041603, 16714041604, 16714044001, 16714044002, 16714044003, 16714044101, 16714044102, 16714044103, 16714046401, 16714046402, 16714046403, 16714046404, 21695040701, 21695068528, 21695077001, 21695085501, 21695085701, 21695099528, 34908062051, 34908062053, 34908062056, 50090015901, 50419040300, 50419040370, 50419040375, 50419040700, 50419040770, 50419040775, 50458017820, 50458019120, 50458019423, 50458019720, 51285012870, 52544005431, 52544008728, 52544016528, 52544016731, 52544022891, 52544038331, 52544038431, 52544055031, 52544055231, 52544055431, 52544084731, 52544098228, 54868404500, 54868424000, 54868474400, 54868477800, 54868485000, 54868503100, 54868592200, 54868593500, 54868604400, 54868610000, 54868616100, 54868616200, 54868621000, 54868627200, 54868627300, 54868627400, 54878727500, 54868627600, 57297087713, 61786038206, 61786038506, 63187005428, 63187045828, 66116043628, 66116047028, 68180083713, 68180086413, 68180086513, 68180086613, 68180088013, 68180090313, 68258500502, 68462013281, 68462065629, 68462065690, 68462065729, 68462065790, 68462067295, 68462071929, 68462072029, 76388028301, 76388028306, 76413010428, 76413010528, 76413011128, 76413011628, 76413011828, 76413012128, 76413012828, 76413013028, 00378728353, 00378728753, 00378729653, 00430053750, 16714007304, 16714035903, 16714036704, 16714040402, 16714040404, 16714040501, 16714040504, 16714040601, 16714040604, 16714040803, 16714041304, 50419040903, 65162031684, 65162034784, 68180087513, 68180087711, 68180087713, 68180088613, 68180089211, 68180089313, 75854060101, 63187074828, 51862010206, 00093542362, 51862003603, 51862003601, 51862026006, 00378730653, 51862002801, 51862054506, 69238153106, 51862002806, 51862009706, 51862031801, 51862031803, 51862000706, 68462013279, 51862027906, 51862027901, 51862056406, 51862056401, 69238155106, 52544029841, 52544029831, 00378729753, 68180087311, 68180087313, 00023586228, 00023586230, 51862001201, 51862001206, 51862029201, 51862029206, 51862007206, 51862023803, 51862051006, 51862047006, 51862047106, 63187075428, 68180083811, 68180083813, 51862028403, 51862004591, 00378728490, 51862004701, 51862004791, 00062192001, 00062192015, 00062192024, 50458019201, 50458019215, 54569541300, 54868467000, 50458019224, 00378334053, 00052027301, 00052027303, 54569586500, 54868483201, 55887075401, 54868483200, 00052027385, 76413013103, 00027013160, 00027013180, 00062330100, 00062330200, 00062330300, 00062330400, 00062330500, 00062330600, 00062330700, 00062330800, 00062330900, 00062331000, 00062331100, 00062331200, 00062331300, 00062334100, 00062334200, 00062334300, 00062334400, 00062334500, 00062334600, 00062334700, 00062334800, 00062334900, 00062335000, 00062335100, 00062335200, 00062338100, 00062338200, 00062338300, 00062338400, 00062338500, 00062338600, 00062338700, 00062338800, 00062338900, 00062364103, 00062364300, 00234005100, 00234013100, 00234013150, 00234013155, 00234013160, 00234013165, 00234013170, 00234013175, 00234013180, 00234013185, 00234013190, 00234013195, 00234013600, 00234013660, 00234013665, 00234013670, 00234013675, 00234013680, 00234013685, 00234013690, 00234013695, 00396401065, 00396401070, 00396401075, 00396401080	
	**HCPCS**	**CPT**
Include	A4261, A4266, J1050, J7303, J7304, S4993	57170

Dx=Diagnosis codes, Px=Procedure codes; DRG=Diagnosis Related Group codes; CPT=Current Procedural Terminology codes; NDC=National Drug Code codes; HCPCS= Healthcare Common Procedure Coding System codes; LARC=long-acting reversible contraception

© 2020 Dalton VK et al. *JAMA Network Open.*

eTable 2. Model Results for Figure 1 Generation

	**Coefficient (95% CI)**
Claim Year[a]	0.023 (-0.006–0.052)
Post-Period Indicator	
2008-2013	REFERENCE
2014-2018	0.514 (0.230–0.797)
Household Income	
<100% FPL	REFERENCE
100-399% FPL	0.517 (0.419–0.616)
≥400% FPL	0.549 (0.451–0.648)
Interaction: Post-Period Indicator & Claim Year	-0.095 (-0.140—0.051)
Interaction: Post-Period Indicator & Household Income	
2014-2018 & 100-399% FPL	-0.376 (-0.661–0.091)
2014-2018 & ≥400% FPL	-0.662 (-0.947—0.378)
Interaction: Household Income & Claim Year	
100-399% FPL	-0.022 (-0.052–0.007)
≥400% FPL	-0.035 (-0.064—0.006)
Interaction: Post-Period Indicator & Household Income & Claim Year	
2014-2018 & 100-399% FPL	0.066 (0.021–0.111)
2014-2018 & ≥400% FPL	0.119 (0.074–0.164)
Race/ethnicity	
White	REFERENCE
Black	-0.152 (-0.161—0.143)

© 2020 Dalton VK et al. *JAMA Network Open.*

Hispanic	-0.000 (-0.008–0.008)
Asian	0.147 (0.137–0.157)
Unknown/missing	-0.004 (-0.019–0.011)
Insurance plan	
POS	REFERENCE
EPO/HMO	0.045 (0.039–0.051)
PPO	-0.151 (-0.170–-0.131)
Indemnity/Other	0.477 (0.442–0.512)
Region	
Southeast	REFERENCE
Great Lakes/Northern Plains	0.119 (0.112–0.125)
Pacific	-0.076 (-0.084–-0.067)
Northeast	-0.041 (-0.049–-0.032)
Mountain	0.125 (0.116–0.134)
Unknown	-0.320 (-0.386–-0.254)
Age, years	0.006 (0.006–0.006)
Number of dependents	0.129 (0.128–0.131)
Constant Term	-3.927 (-4.026–-3.827)

Model Wald $\chi^2(25)=39110.82$, $p\leq0.001$. The comparative interrupted time series model was fit with a Generalized Estimating Equation specified with a binomial distribution and logit link function. This modeling framework appropriately accounts for repeated observations per woman over the study period and allows for the inclusion of both time-varying and woman-specific characteristics.

[a]Claim year was centered at 2008

FPL=federal poverty level; N. Plains=Northern Plains; POS=point of service; EPO=exclusive provider organization; HMO=health maintenance organization; PPO=preferred provider organization

© 2020 Dalton VK et al. *JAMA Network Open.*

eTable 3. Model Results for Figure 2 Generation

	**Coefficient (95% CI)**
Claim Year[a]	-0.037 (-0.058–-0.015)
Post-Period Indicator	
2008-2013	REFERENCE
2014-2018	0.288 (0.107–0.470)
Household Income	
<100% FPL	REFERENCE
100-399% FPL	0.094 (0.012–0.175)
≥400% FPL	-0.099 (-0.180–0.018)
Interaction: Post-Period Indicator & Claim Year	-0.034 (-0.064–-0.004)
Interaction: Post-Period Indicator & Household Income	
2014-2018 & 100-399% FPL	0.005 (-0.177–0.188)
2014-2018 & ≥400% FPL	-0.070 (-0.253–0.112)
Interaction: Household Income & Claim Year	
100-399% FPL	0.043 (0.022–0.065)
≥400% FPL	0.062 (0.040–0.084)
Interaction: Post-Period Indicator & Household Income & Claim Year	
2014-2018 & 100-399% FPL	-0.014 (-0.044–0.117)
2014-2018 & ≥400% FPL	-0.004 (-0.034–0.026)
Race/ethnicity	
White	REFERENCE
Black	0.291 (0.285–0.297)

© 2020 Dalton VK et al. *JAMA Network Open.*

Hispanic	0.336 (0.331–0.342)
Asian	0.564 (0.555–0.572)
Unknown/missing	0.131 (0.121–0.141)
Insurance plan	
POS	REFERENCE
EPO/HMO	0.061 (0.057–0.065)
PPO	-0.010 (-0.122–0.002)
Indemnity/Other	0.315 (0.285–0.346)
Region	
Southeast	REFERENCE
Great Lakes/Northern Plains	-0.023 (-0.028–-0.019)
Pacific	0.150 (0.144–0.156)
Northeast	-0.054 (-0.060–-0.048)
Mountain	0.125 (0.116–0.134)
Unknown	0.524 (0.471–0.578)
Age, years	0.031 (0.031–0..031)
Number of dependents	0.130 (0.129–0.131)
Constant Term	-0.651 (-0.733–-0.569)

Model Wald $\chi^2(25)$=186900.98, p≤0.001. The comparative interrupted time series model was fit with a Generalized Estimating Equation specified with a binomial distribution and logit link function. This modeling framework appropriately accounts for repeated observations per woman over the study period and allows for the inclusion of both time-varying and woman-specific characteristics.

[a]Claim year was centered at 2008

FPL=federal poverty level; N. Plains=Northern Plains; POS=point of service; EPO=exclusive provider organization; HMO=health maintenance organization; PPO=preferred provider organization

© 2020 Dalton VK et al. *JAMA Network Open.*
